# The Longitudinal Relationships Between Social Isolation, Frailty, and Health Outcomes Among Canadian Older Adults

**DOI:** 10.1093/geroni/igab046.656

**Published:** 2021-12-17

**Authors:** Fereshteh Mehrabi, François Béland

**Affiliations:** 1 School of Public Health (ESPUM), Université de Montréal, Montreal, Quebec, Canada; 2 Université de Montréal, Montreal, Quebec, Canada

## Abstract

Social isolation and frailty are global public health issues that may lead to poor health outcomes. We tested the two following hypotheses: 1) changes in social isolation and frailty are associated with adverse health outcomes over two years, 2) the associations between social isolation and health vary across different levels of frailty. We estimated a series of latent growth models to test our hypotheses using data from the FRéLE longitudinal study among 1643 Canadian community-dwelling older adults aged 65 years and over. Missing data were handled by pattern mixture models with the assumption of missing not at random. We measured social isolation through social participation, social networks, and social support from different social ties. We assessed frailty using Fried’s criteria. Our results revealed that higher frailty at baseline was associated with a higher rate of comorbidity, depression, and cognitive decline over two years. Less social participation at baseline was associated with comorbidity, depression, and changes in cognitive decline. Less social support from friends, children, partner, and family at baseline was associated with comorbidity, cognitive decline, and changes in depression. Fewer contacts with grandchildren were related to cognitive decline over time. The associations of receiving less support from partner with depression and participating less in social activities with comorbidity, depression, and cognitive decline were higher among frail or prefrail than robust older adults over time. This longitudinal study suggests that intimate connectedness and social participation may ameliorate health status in frail older populations, highlighting the importance of age-friendly city policies.

